# Associations between change fatigue and teaching enthusiasm among Shanghai teachers: the roles of teaching motivation and job satisfaction

**DOI:** 10.3389/fpsyg.2026.1925965

**Published:** 2026-10-14

**Authors:** Zhang Ziying, Li Shiyu, Bao Yuxuan, Hao Yipu

**Affiliations:** 1 Wenhua College, Wuhan, China; 2 China University of Geosciences, Wuhan, China; 3 Hubei University, Wuhan, China; 4 School of Education, Central China Normal University, Wuhan, China

**Keywords:** change fatigue, conservation of resources theory, job satisfaction, teaching enthusiasm, teaching motivation

## Abstract

**Introduction:**

In recent years, frequent educational reforms have placed growing demands on teachers and may contribute to change fatigue. Drawing on Conservation of Resources theory, this study examines how change fatigue relates to teaching enthusiasm and whether teaching motivation and job satisfaction serve as independent and chained mediators.

**Methods:**

Data were drawn from Shanghai teachers who participated in the OECD's 2024 Teaching and Learning International Survey (TALIS). Validated TALIS-based scales measured change fatigue, teaching motivation, job satisfaction, and teaching enthusiasm. Confirmatory factor analysis, correlation analysis, structural equation modeling, and bootstrap-based mediation tests were used to examine the hypothesized relationships.

**Results:**

Change fatigue was negatively associated with teaching motivation and job satisfaction. Teaching motivation was positively associated with job satisfaction and teaching enthusiasm, and job satisfaction was positively associated with teaching enthusiasm. Teaching motivation and job satisfaction each showed an independent indirect association and together showed a chain indirect association between change fatigue and teaching enthusiasm. After including the mediators, no direct association was found between change fatigue and teaching enthusiasm; instead, change fatigue was indirectly and negatively related to teaching enthusiasm through teaching motivation and job satisfaction.

**Conclusion:**

These findings suggest that change fatigue is indirectly associated with lower teaching enthusiasm through lower teaching motivation and job satisfaction. Schools and policymakers should manage the pace of reform, provide resource compensation and professional support, and strengthen teachers' motivation and job satisfaction to sustain teaching enthusiasm.

## Introduction

Global education systems are undergoing an unprecedented wave of transformation. The rapid penetration of artificial intelligence (AI) technology, continuous updates to curriculum standards, and the constant evolution of teaching models have placed the teaching profession in an era of “perpetual change” (Chun et al., 2025; Datnow et al., 2025; Rahimi and Oh, 2024). The intensity of educational reform in China is particularly notable—from the full implementation of the “Double Reduction” policy to the accelerated advancement of educational digital transformation, and from the comprehensive rollout of new curriculum standards to practical explorations of AI-enabled education. Teachers are required to continuously update their knowledge structures, adjust their teaching behaviors, and adapt to new role expectations (Sun et al., 2025; Zou and Song, 2025). However, while change brings development opportunities, it also poses ongoing challenges to teachers' psychological resources. When the frequency, intensity, or duration of change exceeds an individual's adaptive capacity, teachers may experience “change fatigue”—a state of psychological resource depletion connected with prolonged exposure to organizational change (Bernerth et al., 2011).

The theoretical significance of change fatigue lies in its revelation of the tension between change and individual psychological resources. Conservation of Resources Theory (COR) posits that individuals possess a fundamental motivation to acquire, preserve, and protect valuable resources; when resources face the threat of loss, actual loss, or a disproportionate return on investment, individuals experience psychological stress and adopt defensive strategies (Hobfoll, 1989; Hobfoll et al., 2018). In educational contexts, every curriculum reform, technological upgrade, or policy adjustment is linked to teachers investing additional cognitive, emotional, and time resources to adapt to new demands. When changes occur in rapid succession without a buffer period, teachers' resource reserves will continue to decline, a pattern associated with a “loss spiral” (Hobfoll, 2001). Change fatigue is precisely the concentrated manifestation of this sustained resource depletion at the psychological level—teachers feel overwhelmed and unable to keep up, which is linked to negative attitudes toward the changes themselves and even toward teaching as a profession.

Teaching enthusiasm is a core emotional dimension of teachers' professional competence, referring to the positive emotional experiences and state of internal engagement teachers exhibit during teaching activities (Burić and Moè, 2020; Kunter et al., 2011). Teaching enthusiasm is not only related to the quality of teachers' instruction but also shows a relationship with students' engagement and learning outcomes through mechanisms of emotional contagion (Keller et al., 2014; Mahler et al., 2018; Xue, 2024). Maintaining and stimulating teachers' teaching enthusiasm is key to ensuring educational quality and preventing teacher attrition (Bardach et al., 2022). However, amid increasingly frequent educational reforms, teaching enthusiasm in China is facing unprecedented threats (Zhang and Ye, 2024). Change fatigue may be emerging as a factor linked to lower teaching enthusiasm among Chinese teachers.

However, through what psychological association pathways might change fatigue be related to teaching enthusiasm? Existing research has primarily focused on the direct associations between change fatigue and negative outcomes such as occupational burnout and turnover intentions (Limon and Nartgün, 2020; Yildizoglu and Cemaloglu, 2023), with insufficient exploration of the intervening variables through which it is connected to positive psychological states. The COR theory suggests that resource depletion does not occur in isolation but may be connected with other psychological resources through resource-depletion associations (Hobfoll, 2011; Halbesleben et al., 2014). Consistent with this view, change fatigue may be linked to teachers' lower motivational resources (teaching motivation), which in turn may be associated with lower affective evaluation resources (job satisfaction), and ultimately with lower teachers' teaching enthusiasm. Whether this pattern of resource-depletion associations is empirically supported has yet to be systematically tested.

Based on the above analysis, this study adopts COR theory as its theoretical framework and focuses on the following core questions: how is change fatigue related to teachers' teaching enthusiasm? Do teaching motivation and job satisfaction show a chain indirect association between change fatigue and teaching enthusiasm? The contributions of this study are as follows: first, it introduces the concept of change fatigue into the context of Chinese teacher research, thereby expanding the analytical dimensions of teacher psychology research; second, based on COR theory, it constructs and tests a model of resource-depletion associations, revealing the multiple indirect association pathways through which change fatigue is associated with lower teaching enthusiasm.

## Literature review and research hypotheses

### Conservation of resources theory

COR theory, proposed by Hobfoll (1989), serves as a crucial theoretical framework for explaining the dynamic changes in an individual's psychological resources under stressful conditions. The core assumption of this theory is that individuals possess a fundamental motivation to acquire, conserve, nurture, and protect the resources they value (Hobfoll et al., 2018). Resources are defined as things that individuals perceive as helpful in achieving their goals, including material resources (e.g., equipment, compensation), contextual resources (e.g., social support, organizational climate), personal characteristic resources (e.g., self-efficacy, optimism), and energy resources (e.g., time, energy) (Halbesleben et al., 2014).

COR theory comprises three core principles. First, the primacy of resource loss: the psychological weight of resource loss on individuals far exceeds that of resource gain; the loss association is stronger and more enduring than the gain relationship. Second, the resource investment principle: individuals must invest existing resources to prevent resource loss, recover from loss, or acquire new resources; when resource reserves are insufficient, investment capacity declines, creating a “loss spiral.” Third, the resource caravan passageways principle: resources do not exist in isolation but are interconnected and flow together as “resource caravans”; the acquisition or loss of one resource is linked to other related resources through resource passageways (Hobfoll, 2001, 2011).

In the field of educational research, COR theory has been widely applied to explain phenomena such as teachers' work-related stress, burnout, and turnover tendencies (Liu, 2024; Sha and Chang, 2025). In this study, COR theory is employed to frame possible association pathways through which change fatigue may be linked to teaching enthusiasm. Change fatigue is essentially a state of sustained resource depletion—teachers continuously expend cognitive, emotional, and time resources in coping with frequent changes, yet fail to receive sufficient resource replenishment, a process associated with a continuous decline in resource reserves (Bernerth et al., 2011). According to the primacy of resource loss, this resource depletion may have a significant negative relationship with teachers' psychological state. According to the resource investment principle, teachers in a state of resource scarcity may show reduced willingness and capacity to invest additional resources, which could manifest as lower teaching motivation. According to the resource caravan passageways principle, lower teaching motivation may be further linked to teachers' overall work experience and emotional evaluations (i.e., job satisfaction) through “resource caravan passageway,” and ultimately with lower teaching enthusiasm. This theoretical logic forms the basis for deriving the hypotheses in this study.

### Change fatigue: concept, measurement, and applicability to educational contexts

The concept of “change fatigue” originates from the field of organizational behavior. Bernerth et al. (2011) define it as “a negative psychological state experienced by organizational members due to prolonged exposure to organizational change, manifested as feelings of weariness, helplessness, and detachment toward change.” Unlike resistance to change, change fatigue reflects more of a passive reaction connected with resource depletion rather than active opposition to the content of the change.

Although change fatigue and general teacher burnout may share overlapping emotional manifestations (e.g., feelings of exhaustion), they differ substantially in triggers, core psychological states, theoretical frameworks, and measurement foci. First, triggers differ: burnout stems from cumulative daily work stressors such as workload, role conflict, and student misbehavior, whereas change fatigue is specifically connected with identifiable organizational change events (e.g., curriculum reform, policy adjustment, technological upgrade). Second, core psychological states differ: burnout centers on emotional exhaustion, depersonalization, and reduced personal accomplishment (Maslach et al., 2001), while change fatigue involves weariness with change, skepticism about change meaning, and a desire for stability. Bernerth et al. (2011) originally defined change fatigue as a passive exhaustion reaction to prolonged organizational change rather than active resistance to its content; this distinction was later validated in educational settings by Limon and Nartgün (2020), who confirmed that teachers' change fatigue is conceptually separate from general occupational burnout. Third, from a Conservation of Resources perspective, change fatigue is conceptualized as a resource-depletion state connected with continuous investment in coping with frequent changes without timely replenishment, whereas burnout is a broader, longer-term correlate of a general job demands–resources imbalance. Finally, measurement foci differ: the TALIS 2024 items explicitly anchor fatigue to “school changes,” whereas general burnout scales (e.g., the Maslach Burnout Inventory) assess work-related emotional states without reference to a specific change context, supporting discriminant validity at the operational level.

The change fatigue measurement tool was first developed by Bernerth et al. (2011) as a unidimensional scale comprising six items, with its reliability and validity validated across multiple cultural contexts. Limon (2020) adapted this scale for the Turkish educational context by revising the items to make them more culturally appropriate and validated its applicability among teachers. Subsequent studies found that change fatigue levels among teachers are significantly higher than those of professionals in other industries and are closely related to factors such as years of teaching experience and the frequency of school-based changes (Yildizoglu and Cemaloglu, 2023). Young teachers with 10 years or less of teaching experience exhibit higher levels of change fatigue, which may be related to their relatively limited experience and resources in coping with change (Limon and Nartgün, 2020; Yildizoglu and Cemaloglu, 2023).

Applying the concept of change fatigue to the Chinese educational context has a solid practical foundation. Over the past decade, China's basic education sector has undergone a series of major transformations, including revisions to curriculum standards, reforms to the student admission system, the integration of information technology, the implementation of the “Double Reduction” policy, and the use of artificial intelligence to enhance teaching. Moreover, many of these changes have occurred in close succession, with requirements continuously escalating (Sun et al., 2025; Zou and Song, 2025). Teachers must not only cope with the pressures of daily teaching but also continuously learn new concepts, master new skills, and adapt to new norms; the burden of change has become a significant component of teachers' occupational stress (Gupta and Zhao, 2024). Against this backdrop, introducing the concept of change fatigue helps to deepen our understanding of the dynamic process of teachers' psychological resource depletion in a changing environment.

### The association between change fatigue and teachers' teaching enthusiasm

Teaching enthusiasm refers to the positive emotions and intrinsic engagement teachers experience during teaching activities, including feelings of joy, satisfaction, and a sense of meaning in the teaching process (Kunter et al., 2011). Kunter et al. (2011) conceptualized teaching enthusiasm in terms of two dimensions: enthusiasm for teaching and enthusiasm for the subject. Enthusiastic teachers demonstrate higher levels of teaching vitality and emotional engagement, which are positively related to both teaching quality and student learning outcomes (Kunter, 2013; Dewaele and Li, 2021).

The primacy of resource loss in COR theory can explain why change fatigue may be negatively associated with teaching enthusiasm. When teachers are in a state of resource depletion from continually coping with change, the emotional energy and cognitive resources they can devote to teaching activities are likely to be significantly reduced (Hobfoll, 2001, 2011). On the one hand, change fatigue consumes the time and energy that teachers could otherwise devote to pedagogical innovation and emotional engagement; on the other hand, the psychological distress accompanying resource depletion may be associated with a lower capacity of teachers to experience positive emotions during teaching activities—even when the activities themselves are valuable, resource-depleted teachers may struggle to derive pleasure and satisfaction from them (Burić and Moè, 2020). Research indicates that excessive workloads and sustained pressure from change are linked to lower teachers' teaching enthusiasm, and teachers may adopt a defensive strategy of “minimal task completion” (Liu and Zhou, 2026). Based on the above analysis, this study proposes:

H1: change fatigue is significantly negatively correlated with teachers' teaching enthusiasm.

### The mediating association of teaching motivation

Teaching motivation refers to the intrinsic psychological force that drives individuals to choose the teaching profession, engage in teaching work, and pursue continuous professional development (Watt and Richardson, 2008). Based on expectancy-value theory, teachers' teaching motivation can be categorized into intrinsic value motivation, personal utility value motivation, and social utility value motivation (Thomson and Palermo, 2018). Within the TALIS framework, teaching motivation is operationalized into two dimensions—personal utility motivation and social utility motivation—reflecting the diverse driving forces behind teachers' engagement in educational work (Calkins et al., 2024). Teaching motivation is a core correlate of teachers' professional development and is positively associated with their teaching behaviors, professional engagement, and professional resilience (Han and Yin, 2016; Thoonen et al., 2011). Research indicates that teachers with high levels of teaching motivation exhibit a greater willingness to innovate in teaching, stronger professional commitment, and a lower likelihood of leaving the profession (Qin et al., 2025; Zakariya and Wardat, 2024). Teaching motivation is also regarded as a key protective factor for teachers in coping with occupational stress, capable of buffering the negative relationship between work-related stress and mental health (Skaalvik and Skaalvik, 2013).

Based on the resource investment principle of COR theory, teaching motivation can be understood as a teacher's psychological inclination to invest limited personal resources in teaching activities. When teachers perceive that resource investment may yield meaningful returns, they are more willing to invest resources and maintain high levels of teaching motivation (Ding et al., 2025). However, change fatigue may be negatively linked to this psychological balance of resource investment. On the one hand, change fatigue is associated with a reduced stock of resources available to teachers for investment. When teachers are already exhausted from coping with change, the residual resources they can allocate to teaching innovation are extremely limited (Limon and Nartgün, 2020). On the other hand, change fatigue may make teachers pessimistic about the expected returns on resource investment—in an environment of frequent change, teachers may feel that no matter how much effort they put in, new demands will soon emerge, a perception associated with a “sense of futility in investment” (Bernerth et al., 2011). According to the resource investment principle, when individuals anticipate that their resource investment will not yield the expected returns, their willingness to invest tends to be lower. Therefore, change fatigue may be associated with lower teaching motivation through two correlated pathways: lower resource reserves and lower return expectations (Dilekçi et al., 2025).

When teaching motivation is lower, teaching enthusiasm may also be lower. Teaching enthusiasm is essentially a positive emotional-motivational composite state whose generation and maintenance require sufficient teaching motivation. Only when teachers possess an intrinsic desire to engage in teaching actively can they experience sustained positive emotions during teaching activities (Burić and Moè, 2020). Research indicates that teaching motivation is consistently associated with teaching passion; teachers with high teaching motivation report significantly higher levels of teaching passion and job satisfaction (Richardson and Watt, 2014). Based on the above analysis, this study proposes:

H2: change fatigue is significantly negatively correlated with teachers' teaching motivation.
H3: teaching motivation is significantly positively correlated with teachers' teaching enthusiasm.
H4: teaching motivation shows an indirect association between change fatigue and teachers' teaching enthusiasm.

### The mediating association of job satisfaction

Teacher job satisfaction refers to teachers' overall evaluation and emotional response to their work environment and the teaching profession itself (Locke, 1976; Skaalvik and Skaalvik, 2017). The TALIS framework distinguishes teacher job satisfaction into two dimensions: work environment satisfaction (satisfaction with the school) and professional satisfaction (satisfaction with the teaching profession itself) (Wang et al., 2020). Factors such as school leadership, relationships with colleagues, and working conditions are associated with the former. At the same time, the latter involves teachers' perceptions of the value of their profession and their emotional responses to their career choice. Teacher job satisfaction is a core indicator of professional wellbeing and is closely related to teachers' professional commitment, work engagement, intention to leave, and teaching quality (Madigan and Kim, 2021; Toropova et al., 2021). Teachers with high job satisfaction are more likely to exhibit positive teaching behaviors and greater professional resilience (Klassen and Chiu, 2010). Conversely, teachers with low job satisfaction face a higher risk of burnout and a greater likelihood of leaving the profession (Skaalvik and Skaalvik, 2020).

From the perspective of COR theory, job satisfaction can be viewed as an emotional evaluation based on the state of resource accumulation. When teachers perceive that resource gains (such as professional growth, leadership recognition, and peer support) exceed resource expenditures (such as time investment, emotional labor, and cognitive strain) in their work, job satisfaction tends to be higher; conversely, when perceived resource expenditures consistently exceed resource gains, job satisfaction tends to be lower (Hobfoll, 2001). Change fatigue is associated with an imbalance in which teachers' resource expenditure consistently exceeds resource acquisition in a change context; they invest substantial resources in responding to change but fail to receive corresponding positive returns, and this may be linked to lower job satisfaction (Yildizoglu and Cemaloglu, 2023; Jang and Kang, 2026).

Lower job satisfaction is further associated with lower teaching enthusiasm. According to the resource caravan passageways principle, there are channels of mutual relationship between psychological resources across different dimensions (Hobfoll, 2011). As a holistic evaluation of work-related emotions, job satisfaction may serve as an emotional context for daily teaching activities. Teachers who report higher overall job satisfaction also tend to report positive emotions in specific teaching activities, and this pattern is linked to higher teaching enthusiasm (Chen, 2007); conversely, lower job satisfaction tends to co-occur with greater difficulty in experiencing positive emotions in specific teaching activities, and teaching enthusiasm may be correspondingly lower. Empirical research has also confirmed that job satisfaction is significantly associated with teaching enthusiasm, with a moderate and significant positive correlation between the two (Burić and Moè, 2020; Kunter et al., 2011). Based on the above analysis, this study proposes:

H5: change fatigue is significantly negatively correlated with teachers' job satisfaction.
H6: job satisfaction is significantly positively correlated with teachers' teaching enthusiasm.
H7: job satisfaction shows an indirect association between change fatigue and teachers' teaching enthusiasm.

### The chain mediating association of teaching motivation and job satisfaction

According to the resource caravan passageways principle of COR theory, different types of resources are linked in a sequential manner, whereby the loss or acquisition of resources may be associated with a chain reaction through interconnected channels (Hobfoll, 2011). Change fatigue may be indirectly associated with lower teaching enthusiasm through a possible resource-depletion chain involving teaching motivation and job satisfaction. Specifically, teaching motivation, as the internal psychological energy driving teachers' engagement in professional activities, is a core component of teachers' professional identity (Richardson and Watt, 2014). When change fatigue is associated with lower teaching motivation, teachers may reduce their proactive engagement and psychological involvement in teaching activities, a pattern that may co-occur with fewer opportunities to experience positive emotions and a sense of accomplishment in their work. These cumulative experiences may be associated with a decline in teachers' overall emotional evaluation of their work—that is, job satisfaction (Judge et al., 2017). Empirical evidence supports this relationship. Research indicates that teaching motivation is significantly associated with teacher job satisfaction, and that teachers with high teaching motivation experience more positive emotions and higher professional well-being in their work (Bogler and Nir, 2015; Richardson and Watt, 2014). Therefore, based on the above analysis, this study proposes:

H8: teaching motivation is significantly positively correlated with job satisfaction.
H9: teaching motivation and job satisfaction show a chain indirect association between change fatigue and teachers' teaching enthusiasm.

After analyzing the relevant literature, we proposed nine research hypotheses. We integrated all hypotheses to construct the following analytical framework (shown in Figure 1).

**Figure 1:**
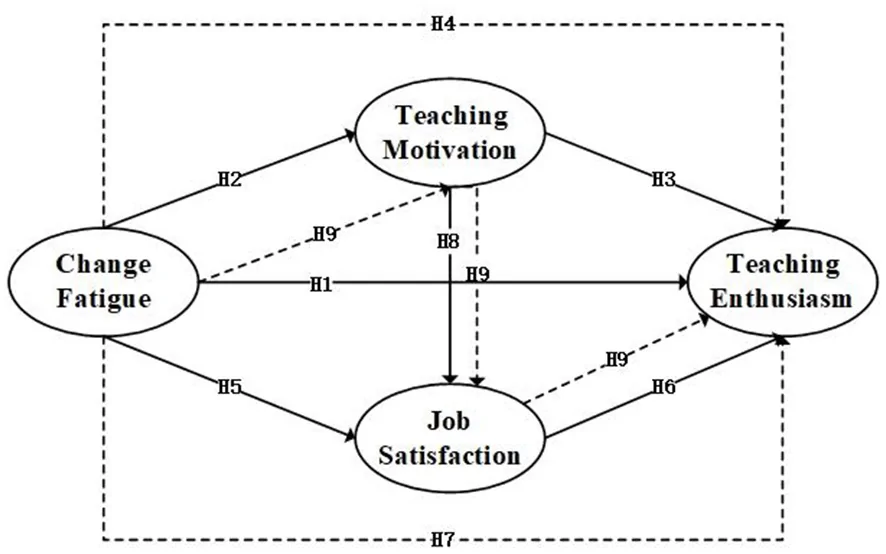
The hypothesized framework.

## Methods

### Data sources and sample

This study utilized data from the Shanghai region of the Organization for Economic Co-operation and Development (OECD)'s 2024 Teaching and Learning International Survey (TALIS 2024). TALIS is the largest global survey of teachers and school principals conducted by the OECD. The 2024 cycle covers 55 education systems, with survey content spanning multiple areas including teacher professional development, teaching practices, working conditions, school climate, and the use of digital technologies.

The selection of the TALIS 2024 Shanghai dataset for this study is appropriate for three reasons. First, TALIS 2024 introduced new core variables such as teacher change fatigue, providing a data foundation for the research. Second, Shanghai teachers demonstrated high survey participation rates and high data quality, and the sample represents the overall situation of junior high school teachers in Shanghai. Third, as a pioneer in China's educational reform, Shanghai has recently experienced the combined effects of multiple transformations—including curriculum reform, digital transformation, and the “Double Reduction” policy—making it a typical context for studying change fatigue (Liang et al., 2016).

TALIS 2024 employs a cyclical survey design; consequently, there are design-related missing values when items are presented to only a portion of the sample (1/3 or 2/3). Because such missing values are considered completely random (MCAR; Organisation for Economic Co-operation Development., 2025), this study utilized cases with no missing data on the primary variable measurement items. The final sample size for this study was 1,329. Table 1 presents the demographic characteristics of the sample. As shown in Table 1, in terms of gender distribution, there were 973 female teachers (73.21%) and 356 male teachers (26.79%), with the proportion of female teachers significantly higher than that of male teachers. In terms of educational background, the vast majority of teachers held a bachelor's degree, totaling 1,033 (77.73%); 292 teachers (21.97%) held a master's or doctoral degree; and only 4 teachers (0.30%) held an associate degree or lower, indicating a relatively high overall educational level within the sample. In terms of years of teaching experience, the largest group consisted of senior teachers with over 20 years of experience, totaling 504 (37.92%); 318 teachers (23.93%) had 11–20 years of experience; 241 teachers (18.13%) had 6–10 years of experience; and 266 were novice teachers with 5 years or less of experience (20.02%).

**Table 1:** Demographic characteristics of the sample (*N* = 1,329).

Variable	Category	*N*	%
Gender	Female	973	73.21
	Male	356	26.79
Educational background	Associate degree or lower	4	0.30
	Bachelor's degree	1,033	77.73
	Graduate degree	292	21.97
Years of teaching experience	5 years or less	266	20.02
	6–10 years	241	18.13
	11–20 years	318	23.93
	Over 20 years	504	37.92

### Measurement

#### Change fatigue

The TALIS 2024 questionnaire drew inspiration from Limon and Nartgün (2020), who adapted Bernerth et al. (2011) Change Fatigue Scale for an educational context. It selected core items reflecting “the depletion of psychological resources connected with continuous change and demands for change” to construct a measure of change fatigue. The scale consists of five items, including the following examples: “I am tired of all the changes at this school” and “I would prefer a period of stability at my school before any changes are made.” All items use a 4-point Likert scale (“1 = Strongly Disagree” to “4 = Strongly Agree”). This method of construct development based on existing items is widely used in international studies using TALIS data; its rationale is that the selected items closely align with the theoretical connotations of the target construct in terms of content validity. In this study, the Cronbach's α coefficient for this scale was 0.819. CFA results indicated acceptable fit: χ^2^/*df* = 6.747, RMSEA = 0.066, CFI = 0.984, TLI = 0.959, SRMR = 0.022, with standardized factor loadings ranging from 0.478 to 0.911. All major fit indices met recommended standards, indicating that the scale effectively measures teacher change fatigue.

#### Teaching enthusiasm

The measurement of teaching enthusiasm draws on the conceptualizations of teaching enthusiasm by Kunter et al. (2011) and Burić and Moè (2020), selecting items from the TALIS 2024 Teacher Work Experiences module that reflect positive emotional engagement and intrinsic enjoyment in teaching. The scale comprises four items, specifically: (1) “I like the subject(s) that I teach” (TT4G80A); (2) “I often feel happy while I teach” (TT4G80B); (3) “I generally teach with enthusiasm” (TT4G80C); (4) “The interesting challenges of teaching give me satisfaction” (TT4G80D). All items use a 4-point Likert scale (1 = Strongly Disagree, 4 = Strongly Agree). This scale has demonstrated good reliability and validity in previous studies (Zhang and Hao, 2026). In this study, the scale's Cronbach's α coefficient was 0.908. CFA results indicated acceptable fit: χ^2^/*df* = 8.052, RMSEA = 0.073, CFI = 0.986, TLI = 0.957, SRMR = 0.017, with standardized factor loadings ranging from 0.782 to 0.881. All major fit indices met recommended standards, indicating that the scale effectively measures teachers' teaching enthusiasm.

#### Teaching motivation

The teaching motivation scale in the TALIS survey consists of two subscales: personal utility motivation and social utility motivation. In the questionnaire, teachers were asked how important personal utility and social utility were as motivations for choosing the teaching profession. Items measuring personal utility motivation include statements such as “Teaching is a secure job.” In contrast, items measuring social utility motivation include statements such as “Teaching allows me to influence the next generation.” All items use a 4-point Likert scale. This method for constructing motivation variables from TALIS items has been validated in prior research (Calkins et al., 2024). The Cronbach's α coefficient for personal utility motivation was 0.762, and for social utility motivation, it was 0.829. The Cronbach's α coefficient for the total scale was 0.863. CFA results indicated acceptable fit: χ^2^/*df* = 8.333, RMSEA = 0.074, CFI = 0.942, TLI = 0.906, SRMR = 0.046, with standardized factor loadings ranging from 0.647 to 0.797. All major fit indices met the recommended standards. The scale used in this study demonstrated acceptable reliability and validity.

#### Job satisfaction

Teacher job satisfaction was measured using the job satisfaction scale from the TALIS 2024 Teacher Questionnaire. This scale consists of 8 items covering teachers' evaluations of their work environment and career satisfaction, such as “I enjoy working at this school” and “The advantages of being a teacher clearly outweigh the disadvantages.” The scale uses a 4-point Likert scale, with higher scores indicating greater teacher job satisfaction. This measurement method is consistent with existing TALIS research (Lopes and Oliveira, 2020). In this study, the Cronbach's α coefficient for this scale was 0.854. CFA results indicated acceptable fit: χ^2^*/df* = 4.700, RMSEA = 0.053, CFI = 0.979, TLI = 0.958, SRMR = 0.026, with standardized factor loadings ranging from 0.438 to 0.890. All major fit indices met recommended standards, indicating that the scale effectively measures teacher job satisfaction.

#### Control variables

Following previous studies (Ting and Jamil, 2026; Wu and Wu, 2026), this study included teachers' gender (0 = male, 1 = female), educational level, years of teaching experience (continuous variable), and school location (rural or urban) as control variables in the model to eliminate potential confounding associations of demographic characteristics on the relationship between the core variables.

### Data analysis methods

This study utilized Stata 17 and Mplus 8.3 for data analysis. The analytical process consisted of the following steps. First, the Harman's single-factor test was employed to assess common method bias. Second, confirmatory factor analysis was conducted to examine the discriminant and convergent validity of each construct, and Cronbach's α coefficients were calculated for each variable. Third, descriptive statistics and correlation analysis were performed to examine the relationships among the variables. Fourth, a structural equation model was used to test the hypothesized model. The Bootstrap method (with 5,000 repetitions) was employed to test the significance of the indirect associations; if the 95% confidence interval did not include zero, the indirect association was considered significant. Model fit was assessed using indicators such as χ^2^*/df*, CFI, TLI, RMSEA, and SRMR, following the recommended criteria of Hu and Bentler (1999): CFI and TLI greater than 0.90, RMSEA less than 0.08, and SRMR less than 0.06 indicate good model fit.

## Results

### Assessment of common method bias and discriminant validity

The data used in this study were derived from teachers' self-reports, thus posing a potential risk of common method bias. To reduce the potential risk of common-method bias, the OECD introduced several procedural mitigation strategies during the development and administration of the TALIS 2024 questionnaire, such as ensuring respondent anonymity, using diverse wording for items, and adopting different scale anchors. At the statistical level, this study employed Harman's single-factor test, incorporating all measurement items for the four core constructs into a single exploratory factor analysis. The results showed that, before rotation, the first factor explained 33.11% of the variance, which is below the 40% critical threshold (Podsakoff et al., 2012), suggesting that common-method bias did not appear to pose a serious threat to the research conclusions.

Second, as shown in Table 2, this study further assessed common method bias and discriminant validity by comparing a series of nested CFA models. In the four-factor model, which specified change fatigue, teaching motivation, job satisfaction, and teaching enthusiasm as independent constructs, the model fit was good: χ^2^*/df* = 4.627, CFI = 0.948, TLI = 0.939, RMSEA = 0.052, and SRMR = 0.056. Compared with other models, the four-factor model fit the data better than the three-factor model, which combined job satisfaction and teaching enthusiasm into a single factor (Δχ^2^ = 1,430.559, Δ*df* = 5, *p* < 0.001), the two-factor model that combined teaching motivation, job satisfaction, and teaching enthusiasm (Δχ^2^ = 4,104.912, Δ*df* = 9, *p* < 0.001), and the single-factor model (Δχ^2^ = 11,242.463, Δ*df* = 10, *p* < 0.001). The single-factor model exhibited extremely poor fit, indicating that the data structure cannot be explained by a single latent factor and providing additional evidence that common-method bias is unlikely to pose a serious concern.

**Table 2:** Confirmatory factor analysis: model comparison for discriminant validity.

Model	*χ^2^*	*df*	*χ^2^/df*	CFI	TLI	RMSEA	SRMR	*Δχ*^2^ (*Δdf*)
Four-factor model (baseline)	1,096.786	237	4.627	0.948	0.939	0.052	0.056	—
Three-factor model (JS+TE merged)	2,527.345	242	10.444	0.861	0.841	0.084	0.077	1,430.559 (5)^***^
Two-factor model (TM+JS+TE merged)	5,201.698	246	21.145	0.698	0.661	0.123	0.105	4,104.912 (9)^***^
One-factor model	12,339.249	247	49.957	0.262	0.176	0.192	0.283	11,242.463 (10)^***^

TM, Teaching Motivation; JS, Job Satisfaction; TE, Teaching Enthusiasm. ^***^p < 0.001.

This study further employed the Heterotrait-Monotrait ratio (HTMT) test to examine discriminant validity. Table 3 presents the HTMT results. As shown in Table 3, the highest HTMT value among all pairwise comparisons was 0.685 (between job satisfaction and teaching enthusiasm), and all values were below the conservative threshold of 0.85 (Henseler et al., 2015). The remaining pairwise values in the HTMT matrix ranged from 0.239 to 0.495, indicating clear discriminant validity among the latent variables and suitability for further structural equation modeling analysis.

**Table 3:** Heterotrait-monotrait ratio test.

Variables	1	2	3
1. Change Fatigue			
2. Teaching Motivation	0.239		
3. Job Satisfaction	0.495	0.445	
4. Teaching Enthusiasm	0.262	0.410	0.685

### Descriptive statistics and correlation analysis

The means, standard deviations, and correlation coefficients for each variable are shown in Table 4. Change fatigue (M = 2.119, SD = 0.511) was significantly and negatively correlated with teaching enthusiasm (*r* = −0.439, *p* < 0.001), as well as with teaching motivation (*r* = −0.282, *p* < 0.001) and job satisfaction (*r* = −0.219, *p* < 0.001). Teaching motivation (M = 3.298, SD = 0.517) was significantly and positively correlated with teaching enthusiasm (*r* = 0.573, *p* < 0.001), and job satisfaction (M = 3.626, SD = 0.427) was significantly and positively correlated with teaching enthusiasm (*r* = 0.397, *p* < 0.001). There was also a significant positive correlation between teaching motivation and job satisfaction (*r* = 0.375, *p* < 0.001). These correlation patterns provided preliminary support for subsequent tests of mediation associations.

**Table 4:** Descriptive statistics and correlation analysis.

Variables	M	SD	1	2	3	4
1. Change Fatigue	2.119	0.511	1			
2. Teaching Motivation	3.298	0.517	−0.282^***^	1		
3. Job Satisfaction	3.626	0.427	−0.219^***^	0.375^***^	1	
4. Teaching Enthusiasm	3.017	0.497	−0.439^***^	0.573^***^	0.397^***^	1

^***^p < 0.001.

### Hypothesis testing

The study used structural equation modeling for hypothesis testing. The model fit indices were as follows: χ^2^/*df* = 3.343, RMSEA = 0.042, CFI = 0.936, TLI = 0.928, SRMR = 0.054. All fit indices fell within acceptable ranges, indicating that the model has a good fit.

In the structural model shown in Figure 2, teaching motivation was significantly negatively correlated with change fatigue (β = −0.245, *p* < 0.001), positively correlated with job satisfaction (β = 0.397, *p* < 0.001), and significantly positively correlated with teaching enthusiasm (β = 0.*140, p* < 0.001). Change fatigue was significantly negatively correlated with job satisfaction (β = −0.356, *p* < 0.001), and there was also a significant positive correlation between job satisfaction and teaching enthusiasm (β = 0.595, *p* < 0.001). However, after including mediating variables, no significant direct relationship was found between change fatigue and teachers' teaching enthusiasm (*p* > 0.05). Therefore, the above results supported H2, H3, H5, H6, and H8. Regarding the indirect association pattern of change fatigue with teaching enthusiasm, further testing is needed to determine whether the chain indirect association of teaching motivation and job satisfaction holds.

**Figure 2:**
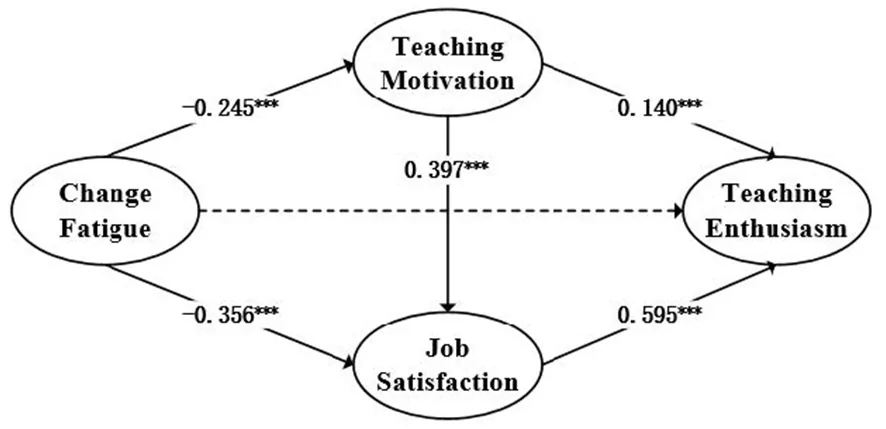
Path coefficients of SEM. ^***^p < 0.001.

To verify the indirect associations of teaching motivation and job satisfaction in the relationship between change fatigue and teaching enthusiasm, the bias-corrected percentile bootstrap method (with 5,000 repetitions) was used to test these associations. The results are shown in Table 5. The total indirect relationship of change fatigue, through the mediating variables, was significant [β = −0.304, SE = 0.032, 95% CI: (−0.367, −0.242)]. Specifically, three indirect paths were identif[ed: (1) The indirect link via teaching motivation (CF → TM → TE) was significant [β = −0.034, SE = 0.010, 95% CI (-0.055, −0.014)]. (2) The indirect link via job satisfaction (JS) (CF → JS → TE) was significant [β = −0.212, SE = 0.031, 95% CI (-0.273, −0.151)]. (3) The chain indirect association via teaching motivation and job satisfaction (CF → TM → JS → TE) was also significant [β = −0.058, SE = 0.009, 95% CI (-0.076, −0.040)]. The 95% confidence intervals for all indirect relationships did not include zero, indicating that teaching motivation and job satisfaction show a chain indirect association between change fatigue and teaching enthusiasm. Therefore, all the above results supported H4, H7, and H9.

**Table 5:** Indirect associations in the SEM.

Path	Estimate	SE	95% CI	
		Lower	Upper
Total indirect relationship	−0.304	0.032	−0.367	−0.242
CF → TM → TE	−0.034	0.010	−0.055	−0.014
CF → JS → TE	−0.212	0.031	−0.273	−0.151
CF → TM → JS → TE	−0.058	0.009	−0.076	−0.040

CF, Change Fatigue; TM, Teaching Motivation; JS, Job Satisfaction; TE, Teaching Enthusiasm.

## Discussion

Based on COR theory, this study examined the relationships between change fatigue and teaching enthusiasm, focusing on the independent and chain indirect associations of teaching motivation and job satisfaction. The findings provide a new theoretical perspective and empirical evidence for understanding the relationship between change fatigue and teaching enthusiasm.

Teaching motivation showed an independent indirect association between change fatigue and teaching enthusiasm. This finding suggests a possible psychological association pathway through which change fatigue is connected with lower teaching enthusiasm: it was associated with lower levels of teachers' internal motivational resources, which in turn was associated with lower positive emotional resources. The indirect relationship of teaching motivation can be better understood through the resource investment principle of COR theory (Hobfoll, 2001). Teaching motivation essentially reflects teachers' psychological inclination to invest personal resources in teaching activities. When teachers perceive that resource investment yields meaningful returns (such as student growth and a sense of teaching accomplishment), they maintain a high willingness to invest; that is, they sustain strong teaching motivation (Ding et al., 2025). However, change fatigue may be associated with lower willingness to invest through correlated pathways. First, change fatigue is associated with a significant consumption of teachers' cognitive and emotional resources, which may correspond to lower “capital” available for investment in teaching. Second, in an environment of frequent change, teachers may feel that no matter how much effort they invest, new demands will soon emerge, and the teaching outcomes generated by previous investments may be “reset” by new changes (Bernerth et al., 2011; Limon and Nartgün, 2020). This “sense of futility” may further be associated with teachers' lower willingness to continue investing resources. A lower level of teaching motivation may co-occur with less emotional and effortful investment in the teaching process and with greater difficulty in experiencing positive teaching enthusiasm.

The study also found that job satisfaction showed a significant independent indirect relationship between change fatigue and teaching enthusiasm. This finding suggests that change fatigue is associated with teaching enthusiasm not only through the internal motivation pathway but also indirectly via the overall job evaluation pathway. Job satisfaction reflects teachers' overall emotional evaluation of their work experience, with a core correlate involving the perceived balance between resource acquisition and resource expenditure (Judge et al., 2017). Change fatigue was associated with a state of imbalance in which resource expenditure consistently exceeds resource acquisition: teachers invest substantial time, energy, and emotional effort in responding to change, yet may not receive commensurate organizational recognition, professional development opportunities, or improvements in teaching outcomes (Yildizoglu and Cemaloglu, 2023). This input-output imbalance was associated with lower levels of teachers' job satisfaction. How might lower job satisfaction be related to lower teaching enthusiasm? According to the resource caravan passageways principle of the COR theory (Hobfoll, 2011), interconnected association channels exist between different types of psychological resources. Job satisfaction, as a diffuse emotional tone of work, provides the emotional context for teachers to engage in specific teaching activities. When job satisfaction is low, this negative emotional tone may permeate teaching activities, which may be associated with teachers' reduced ability to generate and sustain positive emotions in the classroom (Burić and Moè, 2020). Furthermore, according to Affective Events Theory (Qian et al., 2026), an individual's overall work attitude is related to their immediate emotional response to specific work events—teachers with low job satisfaction may find it more difficult to generate positive emotional experiences when facing the same teaching situations. Therefore, change fatigue was indirectly linked to lower levels of teaching enthusiasm for specific teaching activities via its negative association with job satisfaction.

Teaching motivation and job satisfaction showed a chain indirect association between change fatigue and teaching enthusiasm. This finding is consistent with a pattern of resource-depletion associations under conditions of change fatigue: change fatigue was related to lower teaching motivation, lower teaching motivation was related to lower job satisfaction, and lower job satisfaction was related to lower teaching enthusiasm. The observation of this chain indirect association provides important empirical support for the resource caravan passageways principle and the concept of the “resource caravan” in COR theory. Hobfoll (2011) noted that resources do not exist in isolation but are interconnected in the form of a “resource caravan”; the loss of one resource may be connected with other related resources through resource channels, a pattern consistent with resource associations. The chain indirect association pattern observed in this study is consistent with such a pattern of resource associations. The resource depletion connected with change fatigue was related to teachers' core motivational resource—teaching motivation—manifesting as lower internal drive to teach. Lower teaching motivation may co-occur with fewer opportunities to derive positive experiences and a sense of accomplishment from their teaching work. Over time, this may be related to declines in teachers' overall emotional evaluation of their work—a decrease in job satisfaction. Low job satisfaction may further be connected with a negative emotional tone for teaching activities, making it difficult for teachers to mobilize and maintain positive emotions in the classroom, and ultimately may be related to lower teaching enthusiasm.

After incorporating teaching motivation and job satisfaction as mediators, no significant direct relationship was found between change fatigue and teaching enthusiasm, indicating that the direct association was no longer statistically significant after including the indirect pathways in the model. Based on COR theory, change fatigue is a state of exhaustion resulting from the continuous depletion of resources without effective replenishment; it is associated with depletion of emotional and cognitive resources, a pattern that may correspond to a spiral of resource loss. Resource depletion may first be linked to lower teaching motivation and less positive job evaluations, which may correspond to lower teaching motivation and reduced job satisfaction. As a highly arousing positive state, teaching enthusiasm requires a certain level of psychological resources. When resource depletion is related to lower teaching motivation and job satisfaction, teaching enthusiasm may lack the psychological foundation necessary for its maintenance and may thus be indirectly lower. It is evident that change fatigue was not directly associated with teaching enthusiasm but was indirectly and negatively associated with it through lower teaching motivation and job satisfaction. This pattern suggests that the association between change fatigue and lower teaching enthusiasm may be represented by a pattern of relationships: resource depletion, lower motivation and evaluation, and lower enthusiasm. Therefore, maintaining teachers' teaching enthusiasm should not focus solely on reducing change directives; rather, it should involve implementing resource compensation and motivation enhancement during the change process to prevent waning enthusiasm related to diminished motivation and satisfaction. This finding also highlights the importance of considering the complex patterns of association by which change fatigue may be associated with teachers' enthusiasm for teaching, rather than simply assuming a direct relationship.

## Theoretical contributions and practical implications

The theoretical contributions of this study are primarily reflected in two aspects. First, by introducing the concept of change fatigue into research on Chinese teachers' psychology, this study has expanded the analytical dimensions of research on teacher stress. Existing research has primarily focused on teachers' general work stress and occupational burnout, with relatively insufficient attention paid to “change itself as a source of stress.” This study confirms that change fatigue is indirectly associated with teaching enthusiasm through teaching motivation and job satisfaction. Second, based on COR theory, we constructed and tested a model of resource-depletion associations involving change fatigue, teaching motivation, job satisfaction, and teaching enthusiasm, identifying multiple indirect associations consistent with potential indirect association pathways through which change fatigue is associated with lower levels of teachers' positive emotions. The observed indirect associations in this model not only respond to Bernerth et al.'s (2011) call for an in-depth exploration of the mechanisms underlying the consequences of change fatigue but also provide new empirical evidence for the resource caravan passageways principle of COR theory.

The study's findings offer important insights for educational administrators: first, change management should be a core priority for schools. With the observed association between change fatigue and lower teaching enthusiasm, reforms may be more appropriately implemented at a controlled pace, alongside measures such as establishing transition periods, soliciting teacher feedback to enhance engagement, and providing professional training and psychological support. Second, attention to teaching motivation may be relevant where negative associations are observed. Teaching motivation played a significant role in the association between change fatigue and teaching enthusiasm; even in volatile environments, maintaining teaching motivation is associated with higher enthusiasm. Teaching motivation has been associated with practices such as granting teaching autonomy, affirming professional value, and providing growth opportunities (Deci et al., 2017). Third, attention should be paid to the overall improvement of job satisfaction, which is another relevant pathway in the observed associations. Strategies that may be associated with job satisfaction include improving working conditions and compensation, reducing non-teaching burdens, fostering a supportive atmosphere, and establishing fair evaluation mechanisms. Fourth, a monitoring and early warning mechanism for psychological resources may be considered. COR theory suggests that preventive approaches may be more strongly associated with favorable outcomes than repair-oriented approaches; schools might regularly assess teachers' psychological states, identify high-risk groups, and provide timely support such as counseling and professional development opportunities, which may be linked to a lower likelihood of resource depletion.

## Research limitations and future prospects

This study has the following limitations. First, the cross-sectional research design does not allow for strict causal inference among variables. Although theoretical derivations and statistical tests support the directionality of the hypothesized model, there may be a reverse causal relationship or a bidirectional causal relationship between change fatigue and teaching enthusiasm. Future research could adopt a longitudinal tracking design or a quasi-experimental design to more rigorously test causal relationships. Second, all core variables were measured using self-report items from the TALIS 2024 questionnaire, and the study lacked specialized or objective measurement approaches (e.g., validated change-fatigue-specific scales, observational ratings, or administrator reports). Although reliability and validity were acceptable and common-method bias was assessed with no indication that it posed a serious concern, self-report data may still be subject to social desirability and recall bias. Future research should adopt more specialized instruments and triangulate self-reports with objective measures to further validate the findings. Third, this study did not account for the nested structure of teachers within schools; future research could employ multilevel structural equation modeling to examine the associations between variables. Fourth, the sample was limited to the Shanghai region, so caution is warranted when generalizing the study's conclusions to other regions. Shanghai's educational resources and the level of teacher professional development are among the best in the country, and the levels of change fatigue and its underlying association patterns may differ from those in other regions. Future research could conduct cross-regional comparative studies to test the model's cross-context validity.

## Data Availability

The data analyzed in this study are publicly available from the TALIS 2024 Data Repository. The data can be accessed at: https://www.oecd.org/en/data/datasets/talis-2024~database.html.
